# Complete Genome Sequence of a *Pseudomonas putida* Clinical Isolate, Strain H8234

**DOI:** 10.1128/genomeA.00496-13

**Published:** 2013-07-18

**Authors:** Lázaro Molina, Patricia Bernal, Zulema Udaondo, Ana Segura, Juan-Luis Ramos

**Affiliations:** CIDERTA, Laboratorio de Investigación y Control Agroalimentario (LICAH), Parque Huelva Empresarial, Huelva, Spaina; Estación Experimental del Zaidín-CSIC, Granada, Spainb; Universidad de Huelva, Campus de Excelencia Internacional Agroalimentario, Huelva, Spainc

## Abstract

We report the complete genome sequence of *Pseudomonas putida* strain H8234, which was isolated from a hospital patient presenting with bacteremia. This strain has a single chromosome (6,870,827 bp) that contains 6,305 open reading frames. The strain is not a pathogen but exhibits multidrug resistance associated with 40 genomic islands.

## GENOME ANNOUNCEMENT

*Pseudomonas putida* typically inhabits soil and water but is occasionally isolated from patients in hospitals (1, 2). Most of these clinical isolates harbor plasmids carrying genes that encode antibiotic resistance factors (3); these factors can be transferred to pathogens in hospital environments and thus pose a serious threat to public health (4).

*P. putida* H8234 was isolated at the Hospital of Besançon (France) from an inpatient. This strain shows low pathogenic potential compared with *Pseudomonas aeruginosa* PAO1, but it exhibits resistance to commonly used antibiotics. Surprisingly, no plasmid was detected in this strain.

Genomic DNA was purified from strain H8234 using the Wizard Genomic DNA purification kit, sequenced using 454 technology (Macrogen), and assembled into 128 contigs (25× coverage). These contigs were ordered by comparison (BLASTn) with the sequences from other available *P. putida* genomes (accession no. NC_002947.3, CP000712.1, CP000926.1, CP000949.1, and CP002290.1). Genomic gaps were closed by designing primers at the contig ends, followed by PCR and further sequencing of the junction sequences. Genomic DNA was automatically annotated using a program pipeline based on Glimmer 3.0 for gene prediction (5), and BLAST and RPS-BLAST for functional assignment of open reading frames (ORFs) based on sequence similarity to sequences deposited in the NR, SwissProt, COG, Pfam, Smart, and PRK databases (6). Automatic annotations were manually curated.

The single circular chromosome constituting the genome of H8234 is composed of 6,870,827 bases, with a G+C content of 61.6%. It contains 6,305 open reading frames (ORFs), 6 rRNA operons, and 67 tRNA genes. The genome of this strain is remarkably larger than other *P. putida* genomes, which range in size from 5.8 to 6.2 Mb and which contain 5,182 to 5,536 ORFs. The majority of the additional DNA content in the H8234 strain (1.0 Mb) is organized in 44 genetic islands and shows low or no homology to other *P. putida* genomes. Within these islands, we found genes involved in nutrient acquisition: iron uptake (islands VII, XV, XX, XXI, XXIV, XXXIII, XXXVI, and XXXVIII) and transport metabolism of amino acids (islands III, XII, XVII, XXIII, XXIV, XXVI, and XXVIII) and sugars (islands XIV, XXX, XXXI, XXXIII, and XXXV). Also found were genes that encode enzymes to combat host defense responses, including redox balance enzymes (islands XXII, XXIII, XXIV, and XXV), surface antigens (islands XXIV and XXXVII), and damage repair proteins (islands VIII, XXIV, and LX). A broad set of genes in the genetic islands of H8234 are involved in resistance to heavy metals (islands XVIII, XXIII, and XXIV) and aminoglycosides and β-lactam antibiotics (islands XXIII and XXIV). These regions are rich in genes involved in DNA mobilization (transposases, integrases, and conjugational machinery/type IV secretion systems), which may have facilitated the capture of this additional DNA and which may also promote the transmission of genetic material to other microorganisms (islands XXIII, XXIX, and XLII).

The sequenced genome of *P. putida* H8234 serves as a foundation through which we can better understand the evolutionary changes that permit the colonization of human tissues by *P. putida*.

### Nucleotide sequence accession number.

The genome sequence of H8234 was deposited in the GenBank database under the accession no. CP005976.
